# Twin Challenges in Türkiye: Exclusive Breastfeeding Rates and Predictors of Breastfeeding Duration in a Tertiary Care Center

**DOI:** 10.3390/children12060735

**Published:** 2025-06-06

**Authors:** Ayça Kömürlüoğlu, Gökçe Çıplak

**Affiliations:** 1 Department of Pediatrics, Faculty of Medicine, Sivas Cumhuriyet University, Sivas 58140, Türkiye; 2 Department of Neonatology, Faculty of Medicine, Sivas Cumhuriyet University, Sivas 58140, Türkiye; gokceciplak@cumhuriyet.edu.tr

**Keywords:** exclusive breastfeeding, tandem breastfeeding, bottle use, pacifier use, breastfeeding counseling, twin breastfeeding

## Abstract

Objectives: This study aimed to evaluate exclusive breastfeeding (EBF) rates and the duration of breastfeeding among mothers of twins and to identify the maternal, neonatal, and social factors associated with these outcomes. Methods: This retrospective cross-sectional study included 153 mothers of twin infants who were delivered at a tertiary hospital. Data were obtained from medical records and postnatal telephone interviews. Univariate analyses were performed to assess associations with EBF and breastfeeding duration, while multiple linear regression was performed to identify the independent predictors of breastfeeding. Results: The EBF rate within the first six months was 15%, and the mean breastfeeding duration was 10.5 ± 8.3 months. Tandem breastfeeding was positively associated with breastfeeding duration (β = 5.80; 95% CI: 3.51 to 8.10; *p* < 0.001), whereas bottle feeding showed a strong negative association (β = −9.49; 95% CI: −12.88 to −6.10; *p* < 0.001). Infants born before 34 weeks had significantly shorter breastfeeding durations, higher rates of NICU admission and respiratory support, and received less skin-to-skin contact and breastfeeding counselling compared to term infants (*p* < 0.05). Conclusions: Exclusive breastfeeding rates among mothers of twins remain low. Encouraging tandem breastfeeding, reducing bottle use, and providing tailored lactation support—particularly for mothers of preterm infants—may improve breastfeeding outcomes. Breastfeeding support should be adapted according to gestational age in neonatal care.

## 1. Introduction

Breast milk is a unique, natural, and universally recommended source of nutrition that plays a vital role in promoting the healthy growth and development of infants [1]. The World Health Organization (WHO) and the United Nations’ International Children’s Emergency Fund (UNICEF) recommend initiating breastfeeding within the first hour after birth and maintaining exclusive breastfeeding (EBF)—defined as feeding the infant only breast milk, with no additional food or fluids, not even water—for the first six months of life. After six months, appropriate and safe complementary foods should be introduced, while breastfeeding continues until at least two years of age or beyond [2].

The increased use of assisted reproductive technologies (ARTs) has led to a notable increase in twin pregnancies and births globally, including in Türkiye [3]. The global incidence of twin births ranges from 1.5% to 3% of all live births. Twins are considered a high-risk group due to their increased likelihood of preterm birth and associated complications, both perinatal and in the long term [4]. According to data from the Turkish Statistical Institute, the rate of multiple births in Türkiye in 2023 was 3.3%, with 95.6% comprising twin births [5]. Consequently, proper antenatal monitoring, safe delivery practices, and strategies to promote successful breastfeeding in twin pregnancies are of increasing importance. Due to the higher rates of prematurity, low birth weight, and complications involving respiratory and gastrointestinal systems in twins, the benefits of breastfeeding become even more critical [6].

Various challenges such as neonatal intensive care unit (NICU) admission, the delayed initiation of breastfeeding, limited skin-to-skin contact, and increased maternal stress can significantly hinder breastfeeding outcomes in mothers of twins [7]. Breastfeeding in the context of multiple births is a complex psychosocial and physiological process that is shaped by numerous maternal- and infant-related factors. Several studies have consistently demonstrated that mothers of twins have significantly lower rates of exclusive breastfeeding, as well as a reduced initiation and shorter duration of breastfeeding, compared to mothers of singletons, regardless of gestational age [8,9,10]. Factors such as gestational age at birth, birth weight, delivery mode, NICU admission, maternal health status, maternal confidence and intention to breastfeed, and exposure to breastfeeding education have been identified as key determinants of breastfeeding success in twin pregnancies [11,12].

According to the 2018 Turkey Demographic and Health Survey (TDHS 2018) [13], the overall breastfeeding rate in Türkiye was 98%, while the EBF rate for infants under six months was 41%. The global EBF rate was 48% in 2023, with the WHO targeting an increase to over 50% by 2025 [14]. Despite growing interest in improving breastfeeding outcomes, there remains a paucity of data on EBF in twin infants. Reported EBF rates in twin populations vary widely, ranging from 4% to 21.9% across different studies [8,12,15,16,17,18].

Although the international literature provides substantial data on breastfeeding practices among the mothers of twins, particularly from high-income countries, nationally representative studies from Türkiye remain scarce. There is a critical need for context-specific research to understand the unique challenges and influencing factors affecting breastfeeding in twin births within the Turkish healthcare system.

This study aimed to determine the prevalence of exclusive breastfeeding and the average breastfeeding duration among mothers of twins in Türkiye, identify the maternal, neonatal, and social factors associated with these outcomes—including gestational age, NICU admission, and breastfeeding counselling—and provide evidence-based recommendations for clinical practice and health policies. By addressing this gap, this study seeks to contribute to the limited national literature on breastfeeding in relation to multiple births and inform targeted support strategies to improve outcomes in this high-risk group.

## 2. Materials and Methods

### 2.1. Research Design and Study Setting

This retrospective, cross-sectional study was conducted in the Department of Neonatology at Sivas Cumhuriyet University Medical Faculty Hospital. The study population consisted of mothers who delivered live-born twins between 1 January 2018 and 31 December 2023, and whose infants survived. Medical records were reviewed retrospectively, and additional data were obtained through postnatal telephone interviews. Informed consent was secured from all participants before data collection.

### 2.2. Participants and Selection Process

Mothers were eligible for inclusion if they had delivered live-born twins during the study period and met the following criteria: the ability to communicate fluently in Turkish, no significant intellectual or speech impairment, and no history of severe chronic illness or congenital anomalies in the infants. Mothers were excluded if their infants died during or after the neonatal period, if they declined to participate, or if they did not complete the interview.

Out of 168 eligible twin births, 15 were excluded for the following reasons: neonatal death (n = 4), congenital anomalies (n = 3), refusal to participate (n = 5), or incomplete interview (n = 3). A total of 153 mothers were included in the final analysis.

### 2.3. Data Collection Methods

Data were collected using a structured 60-item questionnaire specifically developed for this study, based on a comprehensive review of the existing literature on breastfeeding practices among mothers of twins, including studies by Yokoyama et al. [18] and Kim BY [19] and WHO/UNICEF breastfeeding assessment guidelines [2] (Supplementary File S1). The questionnaire consisted of both open-ended and close-ended items and was administered via telephone interviews conducted by trained researchers.

Interviews were conducted by healthcare professionals who had received standardized training in data collection techniques and neutral probing methods. To minimize recall bias, only the mothers of twins aged ≤7 years were included. Interviewers used concrete reference points—such as hospital discharge and return to work—to improve memory accuracy.

The questionnaire covered the following four main domains: (1) sociodemographic characteristics (e.g., maternal/paternal age, education level, monthly income, and employment status); (2) perinatal and obstetric data (e.g., parity, gestational age, mode of delivery, NICU stay, and birth weight); (3) breastfeeding-related variables (e.g., EBF status, breastfeeding duration, onset of lactation, skin-to-skin contact, and antenatal and postnatal breastfeeding counseling); and (4) maternal experiences and challenges (e.g., nipple problems, breast refusal, milk expression methods, and perceived support systems).

Content validity was evaluated by a panel of three pediatric experts, and several items were revised for clarity and cultural relevance based on their feedback. A pilot test with 10 mothers of twins confirmed the tool’s comprehensibility and practical usability; these pilot test responses were not included in the final analysis. The questionnaire was originally developed and administered in Turkish. For transparency, an English translation is provided (Supplementary File S1); however, this version has not undergone a formal linguistic validation or cross-cultural adaptation process. Since the instrument was designed to capture descriptive data on real-world breastfeeding practices rather than to assess latent constructs, psychometric validation (e.g., factor analysis) was not applicable.

### 2.4. Ethical Aspecst of the Research

This study was approved by the Sivas Cumhuriyet University Non-Interventional Clinical Research Ethics Committee (date:21 March 2024; number: 2024/03-44) and was conducted in accordance with the Declaration of Helsinki. All participants were informed about the purpose, scope, and confidentiality of the research. Verbal informed consent was obtained from each participant at the beginning of the telephone interview. Participation was voluntary, and participants were assured that they could withdraw from the study at any time without consequence. Personal identifiers were not recorded in the dataset. All responses were anonymized and securely stored in encrypted digital files, accessible only to the research team.

### 2.5. Data Analysis

The data analysis was conducted using IBM’s Statistical Package for Social Sciences (SPSS) version 23.0 (SPSS Inc., Chicago, IL, USA). Normally distributed variables were expressed as mean ± standard deviation (SD), while non-normally distributed variables were reported as median (min–max). Categorical variables were presented as frequencies and percentages.

Comparisons between groups were made using the independent samples *t*-test or one-way ANOVA for normally distributed variables, and Mann–Whitney U or Kruskal–Wallis tests for non-normally distributed variables. For multiple comparisons, Tukey’s, Scheffé’s, or Tamhane’s T2 tests were applied based on homogeneity assumptions. The chi-square test was used to evaluate associations between categorical variables.

Binary logistic regression was considered for identifying factors associated with exclusive breastfeeding. However, due to the limited number of EBF cases, this analysis was not feasible; group comparisons were conducted using chi-square and univariate analyses. Multiple linear regression was used to identify the independent predictors of breastfeeding duration. The model included sociodemographic variables (e.g., maternal age, education, employment, and monthly income), perinatal and neonatal characteristics (e.g., mode of delivery, birth weight, NICU admission, and smoking status), and breastfeeding-related behaviors (e.g., bottle and pacifier use, antenatal breastfeeding counseling, breastfeeding initiation time, nipple problems, skin-to-skin contact, and breastfeeding both twins together).

Variables with a *p*-value < 0.20 in univariate analysis were included in multivariate models to enhance model sensitivity and avoid the premature exclusion of potentially relevant predictors, as recommended in observational epidemiological studies. Results were presented as beta coefficients (β) with 95% confidence intervals (95% CI).

Missing data were minimal, and any incomplete responses were clarified via follow-up telephone calls. Observations lacking data for the primary outcomes (e.g., breastfeeding duration or EBF status) were excluded from the final analysis.

### 2.6. Terminology and Study Definitions

All analyses were conducted at both the maternal (n = 153) and children (N = 306) levels, depending on the nature of each variable. In this study, “breastfeeding” includes both direct breastfeeding and feeding with expressed breast milk unless otherwise specified. “Exclusive breastfeeding” is defined as feeding only breast milk with no other liquids or solids, including water, for the first six months of life. Birth weights less than 2500 g were considered as low birth weights (LBW), weights of 2500 grams and above were considered as normal birth weights (NBW), and births before 37 completed weeks of gestation were considered preterm.

## 3. Results

### 3.1. Participants’ Sociodemographic Characteristics

A total of 153 mothers of twins were included in the study. All participants initiated breastfeeding or started a milk supply by pumping at birth. Table 1 presents the sociodemographic characteristics of the study population. The mean maternal age was 34.33 ± 4.67 years, with 81% having completed at least high school education and 69.3% being employed. Nearly half of the mothers were primiparous. Most families lived in urban areas and belonged to middle- or high-income groups.

The gestational age ranged from 27 to 39 weeks, with a mean of 34.5 ± 2.4 weeks, and 92.8% were delivered via cesarean section. While 36.3% of infants had a normal birth weight, 8.2% were classified as having a very low birth weight (<1500 g). NICU admission occurred in 61.4% of cases, with nearly half requiring respiratory support. The median NICU stay was 13.5 days, and the median duration of respiratory support was 5 days.

### 3.2. Breastfeeding Information

The mean breastfeeding duration among mothers of twins was 10.5 ± 8.3 months, and the exclusive breastfeeding (EBF) rate for the first six months was 15%. Breastfeeding initiation within the first hour occurred in only one-third of mothers, and skin-to-skin contact after birth was reported by 44.4% of participants.

While antenatal breastfeeding counseling was received by only 19% of mothers, postnatal counseling was more common (71.9%). Pacifier use was reported in 71.2% of cases, and bottle feeding was reported in 87.6% of cases. On the first day, 24.1% of infants used a pacifier and 34.3% were bottle-fed. By the end of the first month, pacifier and bottle use increased to 56.9% and 76.1%, respectively. Formula feeding was initiated in 88.9% of cases—often on the first day—and primarily based on the decisions of healthcare professionals’.

Breastfeeding difficulties were frequent—54.9% of mothers experienced nipple problems, and 56.2% reported breast refusal, with a median onset of 3.5 months. Most mothers (88.2%) pumped their milk, predominantly using electric breast pumps. Among employed mothers, the median return-to-work time was nine months. Data on the breastfeeding experiences of twin mothers are given in Table 2. 

### 3.3. Twin-Related Factors and Breastfeeding Outcomes

Twins’ gender, birth weight, delivery mode, gestational age, suckling ability at birth, admission to NICU, and need for respiratory support were not significantly associated with exclusive breastfeeding at six months. While gender, delivery type, and birth weight did not affect the mean duration of breastfeeding, shorter durations were observed in infants admitted to the NICU or who received respiratory support. The duration of breastfeeding increased with gestational age, whereby; term infants had a significantly longer duration (*p* = 0.028). Infants with a good suckling ability at birth breastfed for a significantly longer duration than those with poor or no ability (*p* = 0.003). Both EBF rates and breastfeeding duration were significantly lower in infants who used pacifiers or bottles compared to non-users (*p* < 0.05). The results are given in Table 3.

### 3.4. Maternal Factors and Breastfeeding Outcomes

No significant associations were found between maternal age, maternal education level, maternal employment status, parity, smoking status, initiation of breastfeeding time, and exclusive breastfeeding at six months. The average duration of breastfeeding was significantly higher in mothers with a high education level (11.32 ± 8.49 months) compared to those with a lower education level (7 ± 6.68 months; *p* = 0.005), as well as in employed mothers (11.79 ± 8.75 months) compared to unemployed mothers (7.6 ± 6.48 months; *p* = 0.005).

The breastfeeding duration was significantly longer in mothers who never smoked and those who initiated breastfeeding within the first h (*p* = 0.028 and *p* = 0.001, respectively). The results are given in Table 4.

### 3.5. Cultural and Social Factors and Breastfeeding Outcomes

Family type, living area, pregnancy planning, the use of assisted reproductive technologies, husband and family support, and skin-to-skin contact were not significantly associated with either exclusive breastfeeding or breastfeeding duration. However, a higher monthly income was significantly associated with both higher EBF rates and a longer breastfeeding duration (*p* = 0.03 and *p* < 0.01). While antenatal and postnatal breastfeeding counseling did not influence EBF rates, antenatal breastfeeding counseling was associated with a significantly longer breastfeeding duration (*p* = 0.048). Mothers who practiced tandem breastfeeding had significantly higher EBF rates (19% vs. 6.3%) and longer durations (13.1 ± 8.3 vs. 4.8 ± 4.7 months; *p* = 0.040 and *p* < 0.001) (Table 5).

### 3.6. Breastfeeding Outcomes by Gestational Age Group

To assess the impact of gestational age on breastfeeding practices, all neonatal, maternal, and breastfeeding-related parameters were stratified into three gestational age groups, as follows: <34 weeks, 34–36 + 6 weeks (late preterm), and ≥37 weeks (term).

The mean breastfeeding duration increased progressively with gestational maturity, from 7.66 ± 5.16 months in the <34 w group to 12.91 ± 8.53 months in term infants (*p* = 0.013). Although the EBF rate was highest in term infants (21.1%), the difference did not reach statistical significance (*p* = 0.451). Notably, extremely preterm twins (<34 w) had markedly higher NICU admission rates (97.7%) and longer NICU stays (mean 40 ± 21.25 days), compared to 23.7% and 10.7 ± 7.97 days in term infants, respectively (*p* < 0.001). The need for and duration of respiratory support also significantly decreased with increasing gestational age (*p* = 0.001 and *p* = 0.002, respectively). Post hoc analysis confirmed that breastfeeding duration significantly differed between all gestational age groups (*p* < 0.001), with the shortest duration in the <34 w group and the longest in the term infant group.

Breastfeeding-supportive practices such as early initiation (<1 h) and skin-to-skin contact were significantly less common among infants born before 34 weeks (*p* < 0.001 and *p* = 0.004, respectively). While pacifier and bottle use were prevalent across all groups, rates tended to decrease in term infants, although these differences were not statistically significant (*p* > 0.05).

Postnatal breastfeeding counselling was significantly more common among term infants (81.6%) compared to preterm infants (56.8% for <34w) (*p* = 0.026). Breast refusal was most common in the <34w group (68.2%), although this observation was not statistically significant (*p* = 0.162). Tandem breastfeeding rates were comparable across groups. All breastfeeding outcomes according to gestational age group are presented in Table 6.

Despite similar rates of nipple problems and perceived husband/family support across all groups, maternal employment and parental education levels were notably lower in the <34 w group, potentially contributing to suboptimal breastfeeding outcomes. Sociodemographic characteristics according to gestational age group are presented in Supplementary File S2.

### 3.7. Multiple Linear Regression Analysis of Breastfeeding Duration

A multiple linear regression model was constructed to determine the independent predictors of breastfeeding duration. Tandem breastfeeding was strongly and positively associated with a longer breastfeeding duration (β = 5.80; 95% CI: 3.51–8.10; *p* < 0.001). In contrast, bottle feeding was a strong negative predictor (β = −9.49; 95% CI: −12.88 to −6.10; *p* < 0.001). Pacifier use (β = −2.20; 95% CI: −4.59 to 0.18; *p* = 0.072) was marginally associated with a shorter breastfeeding duration.

Variables such as maternal age group, education level, employment status, monthly income, antenatal breastfeeding counseling, nipple problems, the mode of delivery, NICU admission, and birth weight were not significantly associated with breastfeeding duration. The full regression results are presented in Table 7.

## 4. Discussion

Breast milk is the most vital and unique form of nutrition for all infants, especially in multiple pregnancies, where the risks of prematurity and low birth weight are elevated. The birth of twins is associated with a number of challenges, both physical and psychological, when compared to the birth of a single baby. Furthermore, the process of breastfeeding is often hindered by multiple barriers. In this study, EBF rates and breastfeeding durations in twin mothers were determined, and the affecting factors and some solutions to these were discussed.

The EBF rate found in this study was 15%, which is notably lower than rates reported for singleton births; however, it is but consistent with findings from other studies involving twins [8,16,18]. This low rate may be attributed to several factors, including high caesarean section rates, prematurity, NICU admissions, limited breastfeeding counseling, maternal employment, delayed breastfeeding initiation, and the widespread use of pacifiers and bottles —all of which have previously been noted in the literature [20,21,22]. Our findings indicate that EBF was significantly lower in mothers who used pacifiers and bottles and was significantly higher among those who practiced tandem breastfeeding or had higher incomes. Although several factors were found to be associated with EBF in univariate analysis, logistic regression was not performed due to the limited number of EBF cases.

The mean breastfeeding duration was determined to be 10.5 ± 8.3 months. According to Turkey Demographic and Health Survey (TDHS) data, the median breastfeeding duration in the general population increased slightly from 16.5 months in 2013 to 16.7 months in 2018 [13,23]. While national data on breastfeeding in twin pregnancies are lacking, our findings align with other Turkish studies reporting similarly low durations in twin births [24,25]. A twin study in India with a 57.3% prematurity rate found that 46.6% of infants were breastfed for between one and two years [12]. In our study, a shorter breastfeeding duration was associated with low gestational age, NICU admission, pacifier and bottle use, and maternal smoking. Conversely, a longer breastfeeding duration was associated with higher maternal education, higher income, maternal employment, and tandem breastfeeding. Regression analysis identified bottle use and tandem breastfeeding as the strongest independent predictors of breastfeeding duration.

Gestational age is a critical determinant of breastfeeding outcomes, with earlier gestational ages consistently being associated with increased challenges in initiation and maintenance. Infants born preterm, particularly those born before 34 weeks of gestation, are at heightened risk for suboptimal breastfeeding outcomes due to both physiological immaturity (hypotonia, ineffective sucking, etc.) and disrupted perinatal care practices [26,27,28]. Previous studies have underscored this relationship; for instance, Jonsdottir et al. reported significantly lower breastfeeding rates and shorter breastfeeding durations among late preterm twins compared to their term counterparts [11,29]. In our cohort, only 24.8% of infants were born at term, and this group demonstrated significantly longer breastfeeding durations. Our findings further support a strong association between gestational maturity and breastfeeding outcomes, whereby infants born at <34 weeks had shorter breastfeeding durations, had higher rates of breast refusal, and were less likely to benefit from breastfeeding-promoting practices such as early initiation and skin-to-skin contact. These clinical disadvantages are compounded by elevated NICU admission rates and prolonged hospital stays in preterm infants, which may delay the establishment of breastfeeding routines and diminish maternal confidence. Moreover, the lower prevalence of postnatal breastfeeding counseling observed among preterm groups reveals a systemic gap in support services. This is particularly concerning given that structured and consistent lactation support has been shown to significantly improve exclusive breastfeeding rates among preterm infants. In addition, sociodemographic factors such as lower maternal education and parental employment rates in the <34-week group may exacerbate disparities in breastfeeding outcomes, further emphasizing the importance of targeted interventions that address both clinical and social barriers.

It is important to consider the possible influence of the COVID-19 pandemic on breastfeeding practices, particularly among preterm infants who required NICU care. During periods of heightened infection control, many hospitals imposed strict limitations on parental presence in neonatal units, which may have disrupted early maternal–infant contact and delayed the initiation of breastfeeding. Several studies have documented the adverse effects of such restrictions on maternal emotional well-being, lactation, and breastfeeding success [30,31]. Although we observed patterns consistent with the potential impact of COVID-19—such as limited skin-to-skin contact and the delayed initiation of breastfeeding among preterm infants—we did not collect data directly related to COVID-19 restrictions or parental visitation policies during the pandemic. Therefore, our interpretations regarding the pandemic’s influence should be viewed with caution.

The main factors influencing breastfeeding success in twin mothers can be grouped into maternal, infant, and environmental factors. The literature highlights stress, anxiety, perceived milk insufficiency, fatigue, nipple problems, and maternal illness as primary maternal barriers [7,12,32,33]. Premature births; the late initiation of breastfeeding; NICU admissions; poor sucking ability at birth; difficulties in first pumping and then transitioning to breastfeeding in premature babies; not receiving sufficient support from the husband, the family, and healthcare personnel; using pacifiers; and not having knowledge about proper twin breastfeeding techniques were also cited as difficulties [7,32,34,35]. Our study’s open-ended responses identified similar issues, including the difficulty of caring for two babies simultaneously, fatigue, milk insufficiency, and breast-related complications.

Pacifier and bottle use were highly prevalent in our study and were both significantly associated with lower EBF rates and shorter breastfeeding durations. Pacifiers, which are preferred by mothers because they calm babies and make it easier for them to fall asleep, have been shown to shorten the duration of breastfeeding in many studies [36,37,38]. Health professionals should provide guidance on alternative soothing techniques and discourage unnecessary pacifier use. This can help reduce pacifier use and increase breastfeeding rates. Bottle use, which is known to cause nipple confusion, can also lead to breast refusal and early weaning [39]. If necessary, alternative methods to bottle feeding (finger feeding, spoon and cup feeding, etc.) should be suggested to mothers. In a study conducted on multiple pregnancies in Japan, the rate of bottle use was found to be quite high and was associated with low EBF rates [18].

Although skin-to-skin contact was reported in 44.4% of cases, it was not significantly associated with either exclusive breastfeeding or breastfeeding duration in our study. Meta-analyses have shown that postpartum skin-to-skin contact increases mother–infant interaction and promotes higher exclusive breastfeeding rates and overall breastfeeding duration [40,41]. Although skin-to-skin contact is widely recognized to enhance breastfeeding, our study did not confirm this association. The lack of a significant association in our cohort may be attributed to the limited sample size, as well as the timing and context of skin-to-skin practices. In many cases, skin-to-skin contact occurred in NICU settings rather than immediately after delivery—potentially reducing its effectiveness. Additionally, pandemic-related restrictions on maternal presence in NICUs may have disrupted early bonding and delayed lactation initiation. Despite these limitations, given the strong evidence supporting its benefits, we advocate for the routine implementation of immediate skin-to-skin contact following birth, particularly in twin deliveries, as a low-cost and impactful strategy to support breastfeeding success.

Data on the initiation of breastfeeding in twin infants are also limited. The early initiation of breastfeeding was associated with significantly longer breastfeeding durations in our study. In an African study reporting low EBF rates among twins, the rate of early initiation (<1 h) was 24% [8]. In contrast, another study with relatively higher breastfeeding rates in twins reported an early initiation rate of 47.9% [12]. These findings reinforce the importance of early breastfeeding initiation in twin deliveries as a key factor in improving both EBF rates and overall breastfeeding duration. The initiation of breastfeeding, as well as the production of adequate milk, can be facilitated by the provision of systematic support and education from health professionals.

Husband and family support is also very important during the breastfeeding process of twin mothers. Jonsdottir et al. found that support from fathers and grandparents positively influenced breastfeeding behaviour [11], and a meta-analysis confirmed that father-inclusive interventions improve both the initiation and maintenance of breastfeeding [42]. In our study, no significant association was found between husband or family support and either exclusive breastfeeding (EBF) rates or the average breastfeeding duration. However, in light of the data in the literature, we believe that providing twin mothers with support will positively affect breastfeeding processes. Targeted interventions that involve family members could therefore enhance outcomes.

Contrary to popular belief, mothers of multiples have the physiological capacity to produce sufficient breast milk to meet the nutritional needs of all their infants. Breast milk production operates on the principle of supply and demand; thus, frequent and effective breastfeeding stimulates milk synthesis. Various breastfeeding positions are available for the mothers of twins, and infants can be breastfed either separately or simultaneously [43]. Tandem breastfeeding, despite its logistical challenges, is associated with time efficiency, enhanced maternal satisfaction, and potentially improved milk supply. The literature shows that when practiced effectively, tandem feeding can stimulate let-down reflexes and compensate for one infant’s weaker sucking [7,12,43]. However, it requires guidance and support, particularly in the early postpartum period. In a study from Kodinhi, India, only 16.4% of mothers used tandem feeding due to perceived difficulty [12]. In contrast, 68.6% of mothers in our study reported practicing tandem breastfeeding, which was associated with higher EBF rates and longer breastfeeding durations.

We observed a high rate of breast refusal (56.2%), with a median onset at 3.5 months, which may be attributed to the widespread use of pacifiers and bottles. In a national study examining breast refusal, the rate was reported as 35.6% and was associated with sociodemographic factors, levels of social support, and maternal characteristics. Among the identified contributors, pacifier and bottle use were the most significant factors impeding the return to direct breastfeeding [44]. In our study, approximately one-third of mothers were unable to reintroduce their infants to the breast and continued lactation by expressing milk.

Nipple problems also restrict breastfeeding in mothers. More than half of the mothers (54.9%) experienced nipple problems, such as fissures, engorgement, or mastitis. Although our study concluded that it did not affect EBF and the average breastfeeding duration, it is important and necessary for mothers to learn the correct breastfeeding techniques and protect themselves from nipple fissures and complications in order to continue breastfeeding [16]. Breastfeeding counseling is very important and necessary in the case of twin births.

We found that mothers who received antenatal breastfeeding counseling breastfed their infants for significantly longer durations. While Mikami et al. reported that antenatal counseling was associated with improved short-term breastfeeding continuation but had no long-term effect [45], meta-analyses have suggested that breastfeeding support and education enhance breastfeeding duration among healthy term infants. However, there is currently insufficient evidence regarding the effectiveness of such interventions for women with twin or higher-order multiple pregnancies [46]. We believe that the lack of large-scale studies on breastfeeding among mothers of twins limits the generalizability of the existing meta-analytic findings. Therefore, antenatal breastfeeding counseling should be considered essential—not only to prepare mothers for the demands of twin infant care, but also to enhance their motivation and confidence in breastfeeding.

Our study also revealed that smoking mothers had significantly shorter breastfeeding durations compared to non-smoking mothers. Previous studies have similarly reported that smoking negatively impacts breastfeeding duration, with the effect likely being influenced by both the intensity and duration of tobacco use [47,48]. Accordingly, integrating breastfeeding counseling with smoking cessation interventions may represent a valuable strategy to support prolonged breastfeeding among smoking mothers.

In our study, higher maternal education and employment status were positively associated with exclusive breastfeeding and a longer breastfeeding duration. Similarly, a twin study by Wang et al. reported that maternal age, education, and income significantly influenced breastfeeding outcomes [17]. A study from Saudi Arabia reported low exclusive breastfeeding rates in the mothers of twins, with socioeconomic factors playing a key role [15]. These consistent findings may reflect the role of health literacy and access to breastfeeding support among mothers with a higher socioeconomic status. An analysis of 81 low- and middle-income countries from 2000 to 2019 found significant increases in EBF rates among mothers with higher levels of education [49]. These consistent findings suggest that mothers with higher socioeconomic status may be more successful in breastfeeding practices due to better health literacy and access to breastfeeding support.

Breastfeeding twins presents both physical and emotional challenges for mothers, as confirmed by our study, which found low exclusive breastfeeding (EBF) rates and a mean breastfeeding duration of 10.5 months among the mothers of twins. The frequent need to feed two infants at once can lead to increased fatigue and emotional stress, as mothers face heightened demands. Furthermore, preterm birth, which is common in twin pregnancies, complicates the breastfeeding process due to underdeveloped sucking and swallowing reflexes in infants. This can lead to more difficulties and increased stress for mothers [46]. Our findings indicate that providing tailored support, including antenatal and postnatal breastfeeding counseling, is essential to improving breastfeeding outcomes. Programmes that focus on enhancing maternal education, limiting bottle and pacifier use, and encouraging early breastfeeding initiation can help alleviate some of these challenges. Moreover, the mothers of twins are at an increased risk for postpartum depression and anxiety, both of which have been associated with reduced breastfeeding initiation and shorter breastfeeding durations [50]. While our study did not include a direct assessment of maternal mental health, the existing literature strongly supports the association between psychological well-being and breastfeeding outcomes in mothers of twins. Multidisciplinary care can help mothers of twins overcome these challenges, ultimately improving both breastfeeding outcomes and maternal well-being.

It is recommended that breastfeeding be encouraged from the earliest possible moment in order to improve the rates of breastfeeding among mothers of twins. Furthermore, an individualized approach that addresses the factors affecting breastfeeding for each newborn may help improve breastfeeding rates during hospitalization. The mothers of multiple babies should be supported by health professionals in the knowledge that the amount of milk they produce will be sufficient to feed each of their babies, and their self-confidence should be increased in this regard. The absence of breastfeeding support from health professionals was identified as a significant factor contributing to unfavourable breastfeeding outcomes [11].

To improve breastfeeding outcomes in twin mothers, individualized approaches should be prioritized, including early initiation, tandem feeding support, and counseling on non-nutritive sucking practices. Mothers should be reassured that they can physiologically produce enough milk for two babies, and practical support should be offered to build their confidence. Health professionals play a critical role in promoting breastfeeding by providing comprehensive antenatal and postnatal education, discouraging unnecessary formula use, and advocating for breastfeeding-friendly policies within NICUs [16]. Supportive practices such as kangaroo care and early skin-to-skin contact should be standard. At-risk mothers, such as those who are young, primiparous, or with a low educational status, should receive targeted interventions [15,32,33]. Providing economic, familial, and social support to twin mothers and increasing their motivation to breastfeed are very important for healthy generations fed breast milk. While this study was conducted in Türkiye, its findings have broader implications for healthcare systems in other middle-income and resource-limited countries. The suboptimal breastfeeding outcomes observed among mothers of twins—particularly those with preterm deliveries—reflect a global need for more inclusive and structured maternal support strategies. Our results underscore the importance of integrating breastfeeding-friendly practices, targeted counseling, and family-centred NICU care not only in Türkiye but also in similar contexts where the care of multiples remains under-resourced. Policymakers and clinicians can use these findings to adapt scalable interventions that improve breastfeeding outcomes in high-risk populations.

### Strengths and Limitations

One of the main limitations of this study is its single-center design, conducted at a tertiary hospital, which may limit the generalizability of the findings to other settings or populations. Additionally, data collection via telephone interviews may have introduced recall bias or inaccuracies in self-reported maternal behaviors and experiences. The cross-sectional design also precludes causal inferences.

Despite these limitations, this study has several notable strengths. It includes a relatively large and well-characterized sample of twin births and employs a comprehensive, literature-based questionnaire that captures sociodemographic, perinatal, maternal, and behavioral factors. The inclusion of both open- and close-ended questions enabled a more nuanced and contextual understanding of the breastfeeding challenges that are specific to twin mothers. Importantly, this study addresses a significant gap in the national literature and offers evidence that can inform future research, clinical interventions, and public health strategies to improve breastfeeding outcomes in multiple births.

## 5. Conclusions

This study highlights the notably low exclusive breastfeeding rates among mothers of twins and underscores the multifaceted challenges they face. Breastfeeding in this population is influenced by a complex interplay of maternal, neonatal, and social factors. Our findings show that an earlier gestational age—particularly births before 34 weeks—is significantly associated with a shorter breastfeeding duration, increased rates of breast refusal, and a lower exposure to supportive practices such as early initiation and skin-to-skin contact. These challenges are exacerbated by higher NICU admission rates, prolonged hospital stays, and reduced access to postnatal breastfeeding counseling for preterm infants.

To improve EBF rates and breastfeeding duration in twin births, a comprehensive and targeted approach is needed. This should include routine antenatal and postnatal breastfeeding counseling, early initiation protocols, hospital-based lactation consultancy, peer support networks, and breastfeeding-friendly NICU practices. Special attention should be given to the mothers of preterm twins, who face the greatest barriers to breastfeeding success.

Healthcare professionals and policymakers must collaborate to develop inclusive, evidence-based interventions—particularly targeting preterm twin infants cared for in tertiary neonatal units—that address both clinical vulnerabilities and socioeconomic disparities. Strengthening the support systems for at-risk mothers may be crucial in reducing early breastfeeding cessation and improving long-term neonatal health outcomes.

## Figures and Tables

**Table 1:** Participants’ sociodemographic characteristics (n = 153 twin mothers; N = 306 children).

Variable	
Child age (year) *	3.44 ± 1.82
**Child age group N (%)**	
1–2 years	112 (36.6)
3–4 years	106 (34.6)
≥5 years	88 (28.8)
**Child gender N (%)**	
Female	133 (43.7)
Male	173 (56.3)
Mother’s age (year) *	34.33 ± 4.67
**Mother’s age group n (%)**	
<35 years	84 (54.9)
≥35 years	69 (45.1)
**Mother’s education level n (%)**	
<High school	29 (19)
≥High school and higher	124 (81)
**Mother’s employment n (%)**	
Unemployed	47 (30.7)
Employed	106 (69.3)
Father’s age (year) *	37.14 ± 5.24
**Father’s age group n (%)**	
<35 years	48 (31.4)
≥35 years	105 (68.6)
**Father’s education level n (%)**	
<High school	7 (4.6)
≥High school and higher	146 (95.4)
**Father’s employment n (%)**	
Officer	39 (25.5)
Worker	19 (12.4)
Health professional	33 (21.6)
Freelance	20 (13.1)
Others	42 (27.5)
**Mother’s Parity n (%)**	
Primiparous	75 (49)
Multiparous	78 (51)
**Household income n (%)**	
Low income	20 (13.1)
Middle income	65 (42.5)
High income	68 (44.4)
**Living in n (%)**	
Urban	111 (72.5)
Rural	42 (27.5)
**Family type n (%)**	
Nuclear	132 (86.3)
Extended	21 (13.7)
**Twins’ Gestational Age N (%)**	
<34 w	88 (28.8)
34–36 + 6 w (late preterm)	142 (46.4)
≥37 w (term)	76 (24.8)
**Delivery type n (%)**	
Vaginal birth	11 (7.2)
Cesarean section	142 (92.8)
Twins’ Birth Weight (g) *	2274 ± 544
**Twins’ Birth Weight Group N (%)**	
<1500 g	25 (8.2)
1500–2499 g	170 (55.5)
≥2500 g	111 (36.3)
**ART use n (%)**	60 (39.2)
**Planned pregnancy n (%)**	126 (82.4)
**Admission into NICU N (%)**	188 (61.4)
**Respiratory support N (%)**	156 (51)
NICU stay (day) **	13.5 (min 1, max 98)
Respiratory support (day) **	5 (min 1, max 85)

* mean ± SD; ** median (min-max). SD: standard deviation; NICU: neonatal intensive care unit; w: week; ART: assisted reproductive technology. Note: n refers to the number of twin mothers (n = 153), and N refers to the number of twin children (N = 306).

**Table 2:** Breastfeeding information of twins and mothers (n = 153 twin mothers; N = 306 children).

Variable	
Duration of breastfeeding (month) *	10.5 ± 8.3
**Exclusive breastfeeding N (%)**	46 (15)
**Initiation of breastfeeding N (%)**	
<1 h	106 (34.6)
1–24 h	64 (20.9)
≥24 h	136 (44.4)
**Perceived onset of lactation n(%)**	
1st day	77 (50.3)
2nd day	39 (25.5)
3rd day and after	37 (24.2)
**Formula feeding N (%)**	272 (88.9)
**Formula starting time N (%)**	
1st day	130 (47.8)
2–7 days	44 (16.2)
1–4 weeks	38 (14)
2–3 months	38 (14)
4–5 months	10 (3.6)
≥6 months	8 (4.4)
**Initiator of formula feeding N (%)**	
Mother	68 (25)
Health professional (doctor, nurse, lactation consultant, etc.)	200 (73.5)
Others (family, etc.)	4 (1.5)
**Antenatal breastfeeding counseling n (%)**	29 (19)
**Postnatal breastfeeding counseling n (%)**	110 (71.9)
**Skin-to-skin contact at birth N (%)**	136 (44.4)
**Tandem breastfeeding n (%)**	105 (68.6)
**Husband support n (%)**	120 (78.4)
**Family support n (%)**	
No	23 (15)
Yes, but not enough	47 (30.7)
Enough	83 (54.2)
**Nipple problem n (%)**	84 (54.9)
Engorgement	38 (45.2)
Nipple fissure	50 (60.7)
Mastitis	37 (44)
Breast abscess	6 (7.1)
Others	8 (9.5)
**Pumping breast milk n (%)**	135 (88.2)
**Methods of breast milk pumping n (%)**	
Hand expression	25 (18.5)
Manual pumping	30 (22.2)
Single/double electric pumping	99 (73.3)
Hospital-grade pumping	40 (29.6)
Others	4 (3)
**Breast refusal N (%)**	172 (56.2)
Breast refusal time (month) **	3.5
Return-to-work time among employed mothers (month) **	9
**Pacifier use N (%)**	218 (71.2)
**Bottle use N (%)**	268 (87.6)
**Total**	
**n (%)**	153 (100)
**N (%)**	306 (100)

* mean ± SD; ** median. SD: standard deviation. h: hour. Note: n refers to the number of twin mothers (n = 153), and N refers to the number of twin children (N = 306).

**Table 3:** Twin factors associated with exclusive breastfeeding and duration of breastfeeding (n = 153 twin mothers; N = 306 children).

Variable	Exclusive Breastfeeding		*p*-Value	Duration of Breastfeeding (Months) (Mean ± SD)	*p*-Value
	Yes N/n(%)	No N/n(%)			
**Gender**					
Girl (N = 133)	21 (15.8)	112 (84.2)	0.281	10.95 ± 8.63	0.554
Boy (N = 173)	25 (14.5)	148 (85.5)		10.18 ± 8.14	
**Gestational age**					
<34 w (N = 88)	10 (11.4)	78 (88.6)	0.451	7.66 ± 5.16	**0.028**
34–36 + 6 (late preterm) (N = 142)	20 (14.1)	122 (85.9)		10.97 ± 9.35	
≥37 w (term) (N = 76)	16 (21.1)	60 (78.9)		12.91 ± 8.53	
**Delivery type**					
Vaginal birth (n = 11)	1 (9.1)	10 (90.9)	0.567	14.91 ± 8.06	0.054
Cesarean section (n = 142)	22 (15.5)	120 (84.5)		10.16 ± 8.28	
**Birth weight (1st twin)**					
LBW (n = 96)	13 (13.5)	83 (86.5)	0.503	10.1 ± 8.02	0.511
NBW (n = 57)	10 (17.5)	47 (82.5)		11.07 ± 8.86	
**Birth weight (2nd twin)**					
LBW (n = 99)	12 (12.1)	87 (87.9)	0.172	9.74 ± 7.63	0.156
NBW (n = 54)	11 (20.4)	43 (79.6)		11.87 ± 9.39	
**Admission into NICU**					
Yes (N = 188)	30 (16)	158 (84)	0.686	8.96 ± 7.12	**0.015**
No (N = 118)	16 (13.6)	102 (86.4)		12.95 ± 9.53	
**Respiratory support**					
Yes (N = 156)	26 (16.7)	130 (83.3)	0.564	8.87 ± 7.09	**0.029**
No (N = 150)	20 (13.3)	130 (86.7)		12.2 ± 9.19	
**Suckling ability at birth**					
No (N = 88)	18 (20.5)	70 (79.5)	0.480	7.65 ± 6.64	**0.003**
Poor (N = 166)	22 (13.3)	144 (86.7)		9.6 ± 7.79	
Normal (N = 52)	6 (11.5)	46 (88.5)		13.87 ± 9.25	
**Pacifier use**					
Yes (N = 218)	22 (10.1)	196 (89.9)	**0.007**	8.72 ± 7.41	**<0.001**
No (N = 88)	24 (27.3)	64 (72.7)		14.91 ± 8.93	
**Bottle use**					
Yes (N = 268)	22 (8.2)	246 (91.8)	**<0.001**	8.81 ± 6.75	**<0.001**
No (N = 38)	24 (63.2)	14 (36.8)		22.42 ± 8.83	

LBW: low birth weight; NBW: normal birth weight; NICU: neonatal intensive care unit; w: week. Note: n refers to the number of twin mothers (n = 153), and N refers to the number of twin children (N = 306).

**Table 4:** Maternal factors associated with exclusive breastfeeding and duration of breastfeeding (n = 153 twin mothers; N = 306 children).

Variable	Exclusive Breastfeeding		*p*-Value	Duration of Breastfeeding (Months) (Mean ± SD)	*p*-Value
	Yes N/n(%)	No N/n(%)			
**Mother’s age**			0.775		0.531
<35 years (n = 84)	12 (14.3)	72 (85.7)		9.54 ± 6.7	
≥35 years (n = 69)	11 (15.9)	58 (84.1)		11.67 ± 9.89	
**Mother’s education level**					
<High school (n = 29)	4 (13.8)	25 (86.2)	0.836	7 ± 6.68	**0.005**
≥High school and higher (n = 124)	19 (15.3)	105 (84.7)		11.32 ± 8.49	
**Mother’s employment**					
Housewife (n = 47)	6 (12.8)	41 (87.2)	0.601	7.6 ± 6.48	**0.005**
Employed (n = 106)	17 (16)	89 (84)		11.79 ± 8.75	
**Parity**					
Primiparous (n = 75)	11 (14.7)	64 (85.3)	0.901	11.15 ± 8.86	0.425
Multiparous (n = 78)	12 (15.4)	66 (84.6)		9.87 ± 7.8	
**Initiation of breastfeeding**					
<1 h (N = 106)	14 (13.2)	92 (86.8)	0.718	14.02 ± 9.25	**0.001**
1–24 h (N = 64)	8 (12.5)	56 (87.5)		8.55 ± 7.46	
≥24 h (N = 136)	24 (17.6)	112 (82.4)		8.68 ± 7.09	
**Smoking status**					
Never smoked (n = 111)	19 (17.1)	92 (82.9)	0.430	11.64 ± 8.78	**0.028**
Postpartum smoker (n = 38)	4 (10.5)	34 (89.5)		7.32 ± 5.74	
Always smoked (n = 4)	0	4 (100)		9 ± 5.04	
**Nipple problem**					
Yes (n = 84)	12 (14.3)	72 (85.7)	0.775	9.55 ± 7.99	0.167
No (n = 69)	11 (15.9)	58 (84.1)		11.28 ± 8.57	

Note: n refers to the number of twin mothers (n = 153), and N refers to the number of twin children (N = 306).

**Table 5:** Cultural and social factors associated with exclusive breastfeeding and duration of breastfeeding (n = 153 twin mothers; N = 306 children).

Variable	Exclusive Breastfeeding		*p*-Value	Duration of Breastfeeding (Months) (Mean ± SD)	*p*-Value
	Yes N/n (%)	No N/n (%)			
**Household income**					
Low income (n = 20)	4 (20)	16 (80)	**0.030**	9.30 ± 9.07	**<0.001**
Middle income (n = 65)	4 (6.2)	61 (93.8)		7.45 ± 5.86	
High income (n = 68)	15 (22.1)	53 (77.9)		13.77 ± 9	
**Living in**					
Urban (n = 111)	17 (15.3)	94 (84.7)	0.874	11 ± 8.56	0.197
Rural (n = 42)	6 (14.3)	36 (85.7)		9.17 ± 7.63	
**Family type**					
Nuclear (n = 132)	21 (15.9)	111 (84.1)	0.692	10.07 ± 8.32	0.416
Extended (n = 21)	2 (9.5)	19 (90.5)		11.71 ± 6.92	
**Pregnancy plan**					
Planned (n = 126)	21 (16.7)	105 (83.3)	0.222	10.93 ± 8.49	0.107
Unplanned (n = 27)	2 (7.4)	25 (92.6)		8.48 ± 7.37	
**Using ART**					
Yes (n = 60)	10 (16.7)	50 (83.3)	0.650	11.32 ± 9.32	0.613
No (n = 93)	13 (14)	80 (86)		9.97 ± 7.63	
**Skin-to-skin contact at birth**					
Yes (N = 136)	32 (18.8)	138 (81.2)	0.142	11.02 ± 8.38	0.423
No (N = 170)	14 (10.3)	122 (89.7)		10.08 ± 8.32	
**Antenatal breastfeeding counseling**					
Yes (n = 29)	4 (13.8)	25 (86.2)	0.836	13.38 ± 9.46	**0.048**
No (n = 124)	19 (15.3)	105 (84.7)		9.83 ± 7.94	
**Postnatal breastfeeding counseling**					
Yes (n = 110)	13 (11.8)	97 (88.2)	0.075	10.97 ± 8.43	0.258
No (n = 43)	10 (23.3)	33 (76.7)		9.27 ± 8.03	
**Tandem breastfeeding**					
Yes (n = 105)	20 (19)	85 (81)	**0.040**	13.13 ± 8.30	**<0.001**
No (n = 48)	3 (6.3)	45 (93.8)		4.75 ± 4.74	
**Husband support**					
Yes (n = 120)	19 (15.8)	101 (84.2)	0.59	10.7 ± 8.4	0.471
No (n = 33)	4 (12.1)	29 (87.9)		9.76 ± 8.15	
**Family support**					
No (n = 23)	6 (26.1)	17 (73.9)	0.273	10.17 ± 8.41	0.861
Yes, but not enough (n = 47)	6 (12.8)	41 (87.2)		9.55 ± 6.85	
Enough (n = 83)	11 (13.3)	72 (86.7)		11.13 ± 9.08	

ART: Assisted reproductive technology. Note: n refers to the number of mothers (n = 153), and N refers to the number of twin children (N = 306).

**Table 6:** Breastfeeding outcomes by gestational age group (n = 153 twin mothers; N = 306 children).

Variable	<34 w (n = 44, N = 88)	34–36 + 6 w (Late Preterm) (n = 71, N = 142)	≥37 w (Term) (n = 38, N = 76)	*p*-Value
EBF rate (%)	11.4	14.1	21.1	0.451
Breastfeeding duration (months) ^¶^	7.66 ± 5.16	10.97 ± 9.35	12.91 ± 8.53	**0.013**
NICU admission (%)	97.7	59.2	23.7	**<0.001**
NICU stay (days) ^¶^	40 ± 21.25	22.2 ± 19.65	10.7 ± 7.97	**<0.001**
Respiratory support (%)	86.4	49.3	13.2	**0.001**
Respiratory support (days) ^µ^	7.0	3.0	3.0	**0.002**
Skin-to-skin contact at birth (%)	29.5	42.3	65.8	**0.004**
Early BF initiation (<1 h) (%)	9.1	33.8	65.8	**<0.001**
Pacifier use (%)	77.3	73.2	60.5	0.218
Bottle use (%)	95.5	85.9	81.6	0.139
Tandem breastfeeding (%)	68.2	69.0	68.4	0.995
Antenatal breastfeeding counseling (%)	11.4	23.9	18.4	0.246
Postnatal breastfeeding counseling (%)	56.8	76.1	81.6	**0.026**
Formula use (%)	93.2	88.7	84.2	0.435
Breast refusal (%)	68.2	52.1	50	0.162
Nipple problem (%)	56.8	53.5	55.3	0.941
Husband support (%)	77.3	78.9	78.9	0.976
Family support (%)	45.5	54.9	63.2	0.191

EBF: exclusive breastfeeding; ^¶^ mean ± SD; ^µ^ median; BF: breastfeeding. Note: n refers to the number of twin mothers (n = 153), and N refers to the number of twin children (N = 306).

**Table 7:** Multiple linear regression analysis of factors associated with breastfeeding duration (months).

Variable	β	SE	95% CI	*p*-Value
Maternal age (≥35 vs. <35)	1.60	1.10	−0.56 to 3.75	0.148
Maternal employment (employed vs. unemployed)	1.70	1.43	−1.10 to 4.51	0.236
Maternal education (≥high school vs. <high school)	0.34	1.55	−2.70 to 3.38	0.827
Monthly income (high vs. low)	0.46	0.99	−1.48 to 2.39	0.643
Bottle use (yes vs. no)	−9.49	1.73	−12.88 to −6.10	**<0.001**
Pacifier use (yes vs. no)	−2.20	1.22	−4.59 to 0.18	0.072
Antenatal breastfeeding counseling (yes vs. no)	1.15	1.36	−1.52 to 3.81	0.401
Initiation of breastfeeding (≥1 h vs. <1 h)	−0.45	0.85	−2.12 to 1.21	0.592
Tandem breastfeeding (yes vs. no)	5.80	1.17	3.51 to 8.10	**<0.001**
Nipple problems (yes vs. no)	1.12	1.03	−0.90 to 3.14	0.280
Delivery type (cesarean vs. vaginal)	0.44	2.11	−3.70 to 4.58	0.835
Birth weight (low vs. normal)	−0.11	0.96	−1.99 to 1.76	0.905
NICU admission (yes vs. no)	−1.12	1.50	−4.07 to 1.82	0.456
Smoking status (smoker vs. non-smoker)	−1.43	1.06	−3.49 to 0.64	0.179
Constant	11.60	5.42	0.98 to 22.22	0.034

β: beta coefficient; SE: standard error; CI: confidence interval.

## Data Availability

The anonymized dataset is available from the corresponding author upon reasonable request due to ethical reasons.

## Supplementary Materials

The following supporting information can be downloaded at: https://www.mdpi.com/article/10.3390/children12060735/s1, Supplementary File S1: Twin breastfeeding questionnaire (60-item structured interview form). Supplementary File S2: Sociodemographic characteristics by gestational age group.

## References

[1] Meek J.Y., Noble L., Section on Breastfeeding (2022). Policy statement: Breastfeeding and the use of human milk. Pediatrics.

[2] World Health Organization Breastfeeding. https://www.who.int/health-topics/breastfeeding#tab=tab_2.

[3] Ülkümen B.A., Pala H.G., Çalık E. (2013). İkiz gebeliklerde fetal ve maternal sonuçların değerlendirilmesi. Dokuz Eylül Üniversitesi Tıp Fakültesi Dergisi.

[4] Monden C., Pison G., Smits J. (2021). Twin Peaks: More twinning in humans than ever before. Hum. Reprod..

[5] Türkiye İstatistik Kurumu (2023). Doğum İstatistikleri. https://data.tuik.gov.tr/Bulten/Index?p=Dogum-Istatistikleri-2023-53708.

[6] Kielbratowska B., Cwiek D., Preis K., Malinowski W., Hofman A. (2010). Breastfeeding of twins. Arch. Perinat. Med..

[7] Cinar N.D., Alvur T.M., Kose D., Nemut T. (2013). Breastfeeding twins: A qualitative study. J. Health Popul. Nutr..

[8] Tahiru R., Agbozo F., Garti H., Abubakari A. (2020). Exclusive breastfeeding and associated factors among mothers with twins in the Tamale metropolis. Int. J. Pediatr..

[9] Porta R., Capdevila E., Botet F., Ginovart G., Moliner E., Nicolàs M., Gutiérrez A., Ponce-Taylor J., Verd S. (2019). Breastfeeding disparities between multiples and singletons by NICU discharge. Nutrients.

[10] Dong D., Ru X., Huang X., Sang T., Li S., Wang Y., Feng Q. (2022). A prospective cohort study on lactation status and breastfeeding challenges in mothers giving birth to preterm infants. Int. Breastfeed. J..

[11] Jonsdottir R.B., Flacking R., Jonsdottir H. (2022). Breastfeeding initiation, duration, and experiences of mothers of late preterm twins: A mixed-methods study. Int. Breastfeed. J..

[12] Bhardwaj G., Smitha M.V. (2024). Breastfeeding Practices in the Twin Town of India—A Cross-Sectional Study. Twin Res. Hum. Genet..

[13] Hacettepe Üniversitesi Nüfus Etütleri Enstitüsü (2018). Türkiye Nüfus Ve Sağlık Araştırması (TNSA). https://fs.hacettepe.edu.tr/hips/dosyalar/Ara%C5%9Ft%C4%B1rmalar%20-%20raporlar/2018%20TNSA/TNSA2018_ana_Rapor_compressed.pdf.

[14] UNICEF (2023). Global Breastfeeding Scorecard. https://www.unicef.org/documents/global-breastfeeding-scorecard-2023.

[15] Allihaibi M.M. (2020). Factors associated with failure of exclusive breastfeeding among mothers of twins in Saudi Arabia. World Fam. Med..

[16] Anjarwati N., Waluyanti F.T., Rachmawati I.N. (2019). Exclusive breastfeeding for twin babies and its influencing factors: A study in East Java, Indonesia. Compr. Child Adolesc. Nurs..

[17] Wang S., Li M., Xiang X., Guo X., Peng C., Wang D., Chen Y. (2023). Analysis on the current situation of twin breastfeeding and its influencing factors. Medicine.

[18] Yokoyama Y., Wada S., Sugimoto M., Katayama M., Saito M., Sono J. (2006). Breastfeeding rates among singletons, twins and triplets in Japan: A population-based study. Twin Res. Hum. Genet..

[19] Kim B.Y. (2016). Factors that influence early breastfeeding of singletons and twins in Korea: A retrospective study. Int. Breastfeed. J..

[20] Akın Ö., Erbil N. (2020). Breastfeeding and mode of delivery: A systematic review. Ordu Univ. J. Nurs. Stud..

[21] Aggarwal R., Garg P., Verma M., Bindal P., Aditi A., Kaur I., Rohilla M., Kakkar R. (2024). Decadal trends in the exclusive breastfeeding practices among working Indian mothers: A multi-level analysis. Int. Breastfeed. J..

[22] Alshammari M.B., Haridi H.K. (2021). Prevalence and determinants of exclusive breastfeeding practice among mothers of children aged 6–24 months in Hail, Saudi Arabia. Scientifica.

[23] Hacettepe Üniversitesi Nüfus Etütleri Enstitüsü (2013). Türkiye Nüfus Ve Sağlık Araştırması (TNSA). https://fs.hacettepe.edu.tr/hips/dosyalar/Ara%C5%9Ft%C4%B1rmalar%20-%20raporlar/2013%20tnsa/TNSA_2013_ana_rapor.

[24] Cinar N., Kose D., Alvur M., Dogu O. (2016). Mothers’ attitudes toward feeding twin babies in the first six months of life: A sample from Sakarya, Turkey. Iran. J. Pediatr..

[25] Gültekin B., Karakoç A. (2020). Comparison of Breastfeeding Success and Breastfeeding Self-Efficacy Perception in Singleton and Twin Births. Arch. Health Sci. Res..

[26] Nagulesapillai T., McDonald S.W., Fenton T.R., Mercader H.F.G., Tough S.C. (2013). Breastfeeding Difficulties and Exclusivity Among Late Preterm and Term Infants: Results from the All Our Babies Study. Can. J. Public Health.

[27] Boies E.G., Vaucher Y.E., The Academy of Breastfeeding Medicine (2016). ABM Clinical Protocol #10: Breastfeeding the Late Preterm (34–36 6/7 Weeks of Gestation) and Early Term Infants (37–38 6/7 Weeks of Gestation), Second Revision 2016. Breastfeed. Med..

[28] Flacking R., Tandberg B.S., Niela-Vilén H., Jónsdóttir R.B., Jonas W., Ewald U., Thomson G. (2021). Positive breastfeeding experiences and facil tators in mothers of preterm and low birthweight infants: A meta-ethnographic review. Int. Breastfeed. J..

[29] Jonsdottir R.B., Jonsdottir H., Orlygsdottir B., Flacking R. (2021). A shorter breastfeeding duration in late preterm infants than term infants during the first year. Acta Paediatr..

[30] Hoying R., Badreldin N., Shah M.D., Bolden J.R., Cummings P., Robinson D.T. (2021). Providing Breastfeeding Support During COVID-19: A Survey of Staff Experiences. J. Hum. Lact..

[31] Lubbe W., Niela-Vilén H., Thomson G., Botha E. (2022). Impact of the COVID-19 Pandemic on Breastfeeding Support Services and Women’s Experiences of Breastfeeding: A Review. Int. J. Women’s Health.

[32] Bhardwaj G., Smitha M.V. (2024). A Narrative Review of Strategies to Optimize Breastfeeding Among Mothers of Twins. Cureus.

[33] Östlund Å., Nordström M., Dykes F., Flacking R. (2010). Breastfeeding in preterm and term twins—Maternal factors associated with early cessation: A population-based study. J. Hum. Lact..

[34] Nyqvist K.H. (2002). Breast-feeding in preterm twins: Development of feeding behavior and milk intake during hospital stay and related caregiving practices. J. Pediatr. Nurs..

[35] Cassidy H., Taylor J., Burton A., Owen A. (2024). A qualitative investigation of experiences of breastfeeding twins and multiples. Midwifery.

[36] Çinar N.D. (2004). The advantages and disadvantages of pacifier use. Contemp. Nurse.

[37] Buccini G.D.S., Pérez-Escamilla R., Paulino L.M., Araujo C.L., Venancio S.I. (2017). Pacifier use and interruption of exclusive breastfeeding: Systematic review and meta-analysis. Matern. Child Nutr..

[38] Karabulut E., Yalcin S.S., Ozdemir-Geyik P., Karaağaoğlu E. (2009). Effect of pacifier use on exclusive and any breastfeeding: A meta-analysis. Turk. J. Pediatr..

[39] Zimmerman E., Thompson K. (2015). Clarifying nipple confusion. J. Perinatol..

[40] Karimi F.Z., Miri H.H., Khadivzadeh T., Maleki-Saghooni N. (2020). The effect of mother-infant skin-to-skin contact immediately after birth on exclusive breastfeeding: A systematic review and meta-analysis. J. Turk. Ger. Gynecol. Assoc..

[41] Martínez-Hortelano J.A., Saz-Lara A., González J.L.G., Aguado S.C., Iglesias-Rus L., Martínez-Vizcaíno V., Garrido-Miguel M. (2025). Skin-to-skin contact and breastfeeding after caesarean section: A systematic review and meta-analysis of intervention studies. Int. J. Nurs. Stud..

[42] Zhou S.-S., Lu J., Qin A., Wang Y., Gao W., Li H., Rao L. (2024). The role of paternal support in breastfeeding outcomes: A meta-analytic review. Int. Breastfeed. J..

[43] Flidel-Rimon O., Shinwell E. (2006). Breast feeding twins and high multiples. Arch. Dis. Child. Fetal Neonatal Ed..

[44] Karaburun I.E.G., Yalçın S.S. (2024). Breast refusal: An analysis of frequency, onset timing, recovery status, and their interplay with breastfeeding self-efficacy and maternal depression. BMC Public Health.

[45] Mikami F.C.F., de Lourdes Brizot M., Tase T.H., Saccuman E., Francisco R.P.V., Zugaib M. (2017). Effect of prenatal counseling on breastfeeding rates in mothers of twins. J. Obstet. Gynecol. Neonatal Nurs..

[46] Whitford H.M., Wallis S.K., Dowswell T., West H.M., Renfrew M.J. (2017). Breastfeeding education and support for women with twins or higher order multiples. Cochrane Database Syst. Rev..

[47] Kozachenko J., Kivite-Urtane A., Berzina F., Stolcere I.E., Lazdane G. (2024). The association of longer breastfeeding duration and socioeconomic, pregnancy, childbirth and postpartum characteristics. Medicina.

[48] Brown H.K., Pablo L., Scime N.V., Aker A.M., Dennis C.-L. (2023). Maternal disability and initiation and duration of breastfeeding: Analysis of a Canadian cross-sectional survey. Int. Breastfeed. J..

[49] Neves P.A., Barros A.J., Gatica-Domínguez G., Vaz J.S., Baker P., Lutter C.K. (2021). Maternal education and equity in breastfeeding: Trends and patterns in 81 low-and middle-income countries between 2000 and 2019. Int. J. Equity Health.

[50] Egsgaard S., Bliddal M., Lund L.C., Vigod S.N., Munk-Olsen T. (2025). Risk and timing of postpartum depression in parents of twins compared to parents of singletons. Acta Psychiatr. Scand..

