# Supplementary material for: Twin Challenges in Türkiye: Exclusive Breastfeeding Rates and Predictors of Breastfeeding Duration in a Tertiary Care Center
Source: Children (Basel). 2025 Jun 6;12(6):735. doi: 10.3390/children12060735 (PMC12191896; doi:10.3390/children12060735)
Supplement: Supplementary file 1 [file children-12-00735-s001.zip › Supplementary File S1.pdf]

Twin Breastfeeding Questionnaire (60-item Structured Interview Form)

1. Mother's age: __________

2. Mother's education level: ☐ Primary ☐ High school ☐ University or higher

3. Mother's occupation: __________

4. Mother's marital status: ☐ Married ☐ Single ☐ Divorced

5. Number of pregnancies: __________

6. Number of children: __________

7. Father's age: __________

8. Father's education level: ☐ Primary ☐ High school ☐ University or higher

9. Father's occupation: __________

10. Total number of children living in the household: __________

11. Family type: ☐ Nuclear ☐ Extended ☐ Single-parent

12. Monthly income: ☐ Minimum wage ☐ 2–3 times the minimum wage ☐ 4 times the minimum wage or more

13. Place of residence: ☐ Urban ☐ District/Village

14. Was the pregnancy planned? ☐ Yes ☐ No

15. Gestational week at delivery: __________

16. Date of delivery: __________

17. Place of delivery (hospital name): __________

18. Mode of delivery: ☐ Vaginal ☐ Cesarean section

19. Birth weight of infants:
 - Baby 1: __________ g
 - Baby 2: __________ g

20. Sex of the babies:

☐ Both Girls ☐ Both Boys ☐ One girl, one boy

21. Were assisted reproductive technologies used? ☐ Yes ☐ No

22. Were the babies admitted to the neonatal intensive care unit (NICU)?

Baby 1 ☐ Yes ☐ No

Baby 2 ☐ Yes ☐ No

If yes, number of days: __________

23. Did the babies receive respiratory support?

Baby 1 ☐ Yes ☐ No

Baby 2 ☐ Yes ☐ No
If yes, number of days: __________

24. How many days did the babies stay in NICU?

Baby 1………………………..

Baby 2………………………...

25. Did any of the babies have a permanent disease? If yes, please specify: __________

26. Did you receive breastfeeding education/counselling before birth? ☐ Yes ☐ No

27. Did you have prior breastfeeding experience before the twins? ☐ Yes ☐ No

28. When did you first breastfeed after birth? ☐ Within 1 hour ☐ Between 1–24 hours ☐ After 1 day

29. When did you perceive the onset of lactation? ☐ Day 1 ☐ Day 2 ☐ After 3 days

30. What was the babies' sucking ability after birth? ☐ Strong ☐ Weak ☐ None

31. Did you receive postnatal breastfeeding counselling from healthcare professionals?

☐ Yes ☐ No

32. Was skin-to-skin contact initiated after birth? ☐ Yes ☐ No

33. Did you breastfeed your babies? ☐ Yes ☐ No

34. Did you practice tandem breastfeeding (breastfeeding both babies at the same time)?

☐ Yes ☐ No

35. Did you room-in with the babies after birth? ☐ Yes ☐ No

36. Who assisted you in the care of your babies? ☐ Spouse ☐ Mother ☐ Mother-in-law ☐ Relative ☐ Babysitter ☐ Other: __________

37. Did you receive support from your spouse in baby care and breastfeeding? ☐ Yes ☐ No

38. How would you describe the support you received from your family? ☐ No support ☐ Some support but insufficient ☐ Sufficient support

39. Did you receive help from a lactation consultant? ☐ Yes ☐ No

40. Duration of breastfeeding (in months): ……………………………………………………………

41. Did you provide anything other than breast milk in the first 6 months (formula, water, etc.)? ☐ Yes ☐ No

42. Did you experience any breastfeeding-related breast problems? ☐ Yes ☐ No
If yes, specify:

☐ Engorgement ☐ Nipple cracks ☐ Mastitis ☐ Fungal infection ☐ Other: __________

43. Did you express your breast milk? ☐ Yes ☐ No
If yes, method: ☐ Hand expression ☐ Manual pump ☐ Electric pump ☐ Hospital-grade pump ☐ Other: __________

44. What was your biggest motivation for breastfeeding?

……

45. What was the most challenging aspect of breastfeeding twins?

……

46. Did your babies experience breast refusal? ☐ Yes ☐ No
If yes, when and how did you resolve it? ……………………………………………………………...

47. Did your babies experience any feeding difficulties? ☐ Yes ☐ No

48. Did you use formula? ☐ Yes ☐ No

49. Who made the decision to start formula feeding? ☐ Healthcare professional ☐ Mother ☐ Other family members ☐ Other: ……………………………………………………………………...

50. Did you give your babies anything other than breast milk in the first 6 months (water, milk, complementary food)? ☐ Yes ☐ No

51. When did you first give formula?

☐ First day ☐ Between days 2–7 ☐ Between 1–4 weeks ☐ Between 2–3 months ☐ Between 4–5 months ☐ 6 months or later

52. When did you introduce complementary foods? …………………………………………………

53. Did you use a bottle while feeding your babies? ☐ Yes ☐ No

54. Did you give your babies pacifiers? ☐ Yes ☐ No

55. Maternal smoking status:

☐ Never smoked ☐ Smoked but not during pregnancy ☐ Currently smoking"

56. If employed, when did you return to work? ………………………………………………………

57. Did returning to work affect breastfeeding?

☐ Yes, negatively ☐ No effect

58. Do you think breastfeeding twins is physically and emotionally exhausting?

☐ Yes ☐ No ☐ Partially

59. Do you think you had enough knowledge about breastfeeding twins? ☐ Yes ☐ No

60. What type of support or information would have been most helpful during your breastfeeding experience with twins?...

Note: The questionnaire was originally developed and administered in Turkish. The English version included here has not been psychometrically validated and is intended solely for documentation and reference purposes.
