# Supplementary material for: Twin Challenges in Türkiye: Exclusive Breastfeeding Rates and Predictors of Breastfeeding Duration in a Tertiary Care Center
Source: Children (Basel). 2025 Jun 6;12(6):735. doi: 10.3390/children12060735 (PMC12191896; doi:10.3390/children12060735)
Supplement: Supplementary file 1 [file children-12-00735-s001.zip › Supplementary File S2.pdf]

Supplementary File 2: Sociodemographic Characteristics by Gestational Age Group (n = 153 twin mothers, N=306 children).

**Variable**	**<34w (n=44, N=88)**	**34-36+6w (late preterm) (n=71, N=142)**	**≥37w (term) (n=38, N=76)**	**p-value**
**Mothers' age (year)***	33.54±5.41	34.6±4.39	34.7±4.26	0.415
**Mothers' age group n (%)**				
<35 years	28 (63.6)	41 (57.7)	15 (39.5)	0.073
≥35 years	16 (36.4)	30 (42.3)	23 (60.5)	
**Mothers' education level n (%)**				
<High school	12 (27.3)	11 (15.5)	6 (15.8)	0.249
≥High school and higher	32 (72.7)	60 (84.5)	32 (84.2)	
**Mothers' employment n (%)**				
Unemployed	21 (47.7)	15 (21.1)	11 (28.9)	**0.011***
Employed	23 (52.3)	56 (78.9)	27 (71.1)	
**Father's age (year)***	37.3±5.58	37±5.67	37.2±3.96	0.951
**Father's age group n (%)**				
<35 years	13 (29.5)	28 (39.4)	7 (18.4)	0.075
≥35 years	31 (70.5)	43 (60.6)	31 (81.6)	
**Father's education level n (%)**				
<High school	8 (18.2)	2 (2.8)	1 (2.6)	**0.004***
≥High school and higher	36 (81.8)	69 (97.2)	37 (97.4)	
**Mothers' parity n (%)**				
Primiparous	18 (40.9)	35 (49.3)	22 (57.9)	0.308
Multiparous	26 (59.1)	36 (50.7)	16 (42.1)	
**Child gender N (%)**				
Female	36 (40.9)	70 (42.3)	36 (47.4)	0.599
Male	52 (59.1)	72 (57.7)	40 (52.6)	
**Household income n (%)**				
Low income	9 (20.5)	7 (9.9)	4 (10.5)	**0.016***
Middle income	25 (56.8)	26 (36.6)	14 (36.8)	
High income	10 (22.7)	38 (53.5)	20 (52.6)	
**Family type** (nuclear n%)	41 (93.2)	61 (85.9)	30 (78.9)	0.216
**Living in n (%)**				
Urban	30 (68.2)	52 (73.2)	29 (76.3)	0.701
Rural	14 (31.8)	19 (26.8)	9 (23.7)	
**Delivery type n (%)**				
Vaginal birth	3 (6.8)	6 (8.5)	2 (5.3)	0.823
Caesarean section	41 (93.2)	65 (91.5)	36 (94.7)	
**Twins birth weight (gram) ***	1.84±0.52	2.4±0.49	2.65±0.48	**<0.001***
**Twins birth weight group N (%)**				
<1500 grams	20 (22.7)	0	0	**<0.001***
1500-2499 grams	62 (70.5)	84 (59.2)	26 (34.2)	
≥2500 grams	6 (6.8)	58 (40.8)	50 (65.8)	
**ART use n (%)**	16 (36.4)	26 (36.6)	18 (47.4)	0.494
**Planned pregnancy n (%)**	36 (81.8)	57 (80.3)	33 (86.8)	0.689
**Perceived onset of lactation n (%)**				
1st day	21 (47.7)	37 (52.1)	19 (50)	0.787
2nd day	11 (25)	20 (28.2)	8 (21.1)	
3rd day and after	12 (27.3)	14 (19.7)	11 (28.9)	

**Suckling ability at birth N (%)**				
No<br>Poor<br>Normal	32 (36.4)<br>52 (59.1)<br>4 (4.5)	16 (11.2)<br>88 (62)<br>38 (26.8)	4 (5.3)<br>26 (34.2)<br>46 (60.5)	**<0.001***
**Mothers smoking status n (%)**				
Never smoked<br>Smoker	31 (70.5)<br>13 (29.5)	52 (73.2)<br>19 (26.8)	28 (73.7)<br>10 (26.3)	0.933

*mean ± SD, SD: standard deviation, ART: Assisted reproductive technologies.

Note: n refers to the number of twin mothers (n = 153), and N refers to the number of twin children (N = 306). Infant-level variables are reported using N.
